# Use of technology for public health surveillance reporting: opportunities, challenges and lessons learnt from Kenya

**DOI:** 10.1186/s12889-020-09222-2

**Published:** 2020-07-13

**Authors:** Ian Njeru, David Kareko, Ngina Kisangau, Daniel Langat, Nzisa Liku, George Owiso, Samantha Dolan, Peter Rabinowitz, Daniel Macharia, Chinyere Ekechi, Marc-Alain Widdowson

**Affiliations:** 1 International Training and Education Centre for Health (I-TECH Kenya), Nairobi, Kenya; 2 Ministry of Health Kenya, Nairobi, Kenya; 3 Division of Global Health Protection, Centers for Disease Control and Prevention, Nairobi, Kenya; 4 Division of Global Health Protection, Centers for Disease Control and Prevention, Atlanta, GA USA

**Keywords:** Public health surveillance, Reporting rates, Intervention group, Comparison group

## Abstract

**Background:**

Effective public health surveillance systems are crucial for early detection and response to outbreaks. In 2016, Kenya transitioned its surveillance system from a standalone web-based surveillance system to the more sustainable and integrated District Health Information System 2 (DHIS2). As part of Global Health Security Agenda (GHSA) initiatives in Kenya, training on use of the new system was conducted among surveillance officers. We evaluated the surveillance indicators during the transition period in order to assess the impact of this training on surveillance metrics and identify challenges affecting reporting rates.

**Methods:**

From February to May 2017, we analysed surveillance data for 13 intervention and 13 comparison counties. An intervention county was defined as one that had received refresher training on DHIS2 while a comparison county was one that had not received training. We evaluated the impact of the training by analysing completeness and timeliness of reporting 15 weeks before and 12 weeks after the training. A chi-square test of independence was used to compare the reporting rates between the two groups. A structured questionnaire was administered to the training participants to assess the challenges affecting surveillance reporting.

**Results:**

The average completeness of reporting for the intervention counties increased from 45 to 62%, i.e. by 17 percentage points (95% CI 16.14–17.86) compared to an increase from 49 to 52% for the comparison group, i.e. by 3 percentage points (95% CI 2.23–3.77). The timeliness of reporting increased from 30 to 51%, i.e. by 21 percentage points (95% CI 20.16–21.84) for the intervention group, compared to an increase from 31 to 38% for the comparison group, i.e.by 7 percentage points (95% CI 6.27–7.73). Major challenges for the low reporting rates included lack of budget support from government, lack of airtime for reporting, health workers strike, health facilities not sending surveillance data, use of wrong denominator to calculate reporting rates and surveillance officers having other competing tasks.

**Conclusions:**

Training plays an important role in improving public health surveillance reporting. However, to improve surveillance reporting rates to the desired national targets, other challenges affecting reporting must be identified and addressed accordingly.

## Background

Infectious diseases remain an important public health problem causing up to 63% of all childhood deaths and 48% of all premature deaths globally [1]. Infectious disease outbreaks if not detected and reported early, can rapidly spread and result in high morbidity and mortality [2]. Effective public health surveillance systems can provide timely and accurate information leading to early detection of potential outbreaks and containing them in the local areas [3]. Unfortunately, public health surveillance systems are poorly developed in many low and middle income countries (LMIC) as demonstrated by the recent Ebola outbreak in West Africa which led to devastating consequences in the health and economy of several countries [4, 5].

A systematic approach is required to strengthen public health surveillance systems that can quickly detect and respond to the initial cases of disease outbreaks and other public health emergencies. The key strategy for implementing public health surveillance in the African countries is the Integrated Disease Surveillance and Response (IDSR) strategy which was launched by WHO Afro in 1998 [6]. IDSR is used as one of the tools that help in the implementation of International Health Regulations (IHR) which are legally binding to member countries [7]. IDSR also supports the implementation of the Global Health Security Agenda (GHSA) [8] and the One Health Initiative (OHI) [9, 10] which are used in many countries to strengthen countries’ capacity to prevent, detect, and rapidly respond to infectious diseases and other public health emergencies.

Although many countries have made significant progress in the implementation of IDSR, many challenges still hinder these countries from achieving optimal implementation. Though varied, many challenges are similar across the countries that have evaluated and published their IDSR performance. These challenges include: inadequate financial resources, poor coordination, weak laboratory capacity, poor communication systems, poor supervision, erratic feedback, inadequate training of health workers, lack of IDSR technical guidelines and reporting tools [11–16].

One of the main goals of IDSR implementation is to monitor disease and public health event trends in order to ensure that any unusual disease patterns such as outbreaks are detected quickly, investigated and responded to within the shortest time possible. For this reason, IDSR performance is often evaluated on completeness of reporting (proportion of health facilities and districts reporting) and timeliness of reporting (proportion of reports sent on time) [17, 18].

The IDSR system in Kenya has a total of 36 reportable priority diseases as per the 2nd Edition of IDSR Technical Guidelines adapted in 2012. The diseases are categorized as follows; diseases targeted for elimination, epidemic prone diseases, diseases of public health importance and public health events of international concern under IHR 2005. These priority diseases have varying reporting timelines and requirements. The Kenyan surveillance system requires that some diseases are reported immediately within 24 h, others weekly and others monthly.

Diseases/conditions that must be reported weekly are 23 as follows: Acute Flaccid Paralysis (AFP), Acute haemorrhagic fever syndrome (Ebola, Marburg, Lassa Fever, Crimean-Congo), Acute Jaundice, Adverse events following immunization (AEFI), Anthrax, Cholera, Dengue fever, Diarrhoea with blood (Shigella), Guinea Worm Disease (Dracunculiasis), Malaria, Malnutrition in under 5 years, Measles, Meningococcal Meningitis, Maternal death, Neonatal death, Neonatal tetanus, Plague, Rift Valley Fever, Severe Acute Respiratory Illness (SARI) clusters, Rabies, Typhoid, Yellow fever and Tuberculosis (Lab confirmed multidrug and extremely drug resistant Tuberculosis).

While many countries have migrated from paper-based to electronic IDSR reporting, not much is published about the electronic platforms that different countries are using for IDSR and the challenges that affect use of these platforms. The surveillance reporting system in Kenya remained mainly manual until 2007 when efforts were made to migrate to an Epi Info-based system (desktop system). Districts (now referred to as sub-counties) would compile their reports and send them to the national level via email, fax, courier or hand delivery. The national team would then enter the data into the digital desktop platform (Epi Info) for analysis. A weekly epidemiological bulletin was produced and shared back to the districts via email.

In 2011, the Ministry of Health (MOH) shifted reporting from the Epi Info system to a standalone (not integrated with other program systems) web-based system (also known as e-IDSR) due to challenges such as untimely and incomplete reporting especially from hard to reach areas. In this system, data from health facilities were captured electronically using computers at the sub-county level rather than at national level, while higher levels (national and county) were given rights to view and use the data. In August 2016, the country migrated eIDSR from the standalone system to the District Health Information System (DHIS2). The DHIS2 platform is an integrated web-based platform with capacity to report data from all other programs. System maintenance costs are therefore shared across programs making the system more sustainable.

Before the switch to DHIS2 in August 2016, all 47 county surveillance officers, 304 sub county surveillance officers, and 304 sub-county health records and information officers were trained on selected modules on IDSR surveillance strategy as well as the practical use of the DHIS2 platform. The training was conducted between January and June 2016 and also included facility surveillance focal persons from each of the Level 4 (sub-county), Level 5 (county) and Level 6 (national) health facilities.

**Fig. 1:**
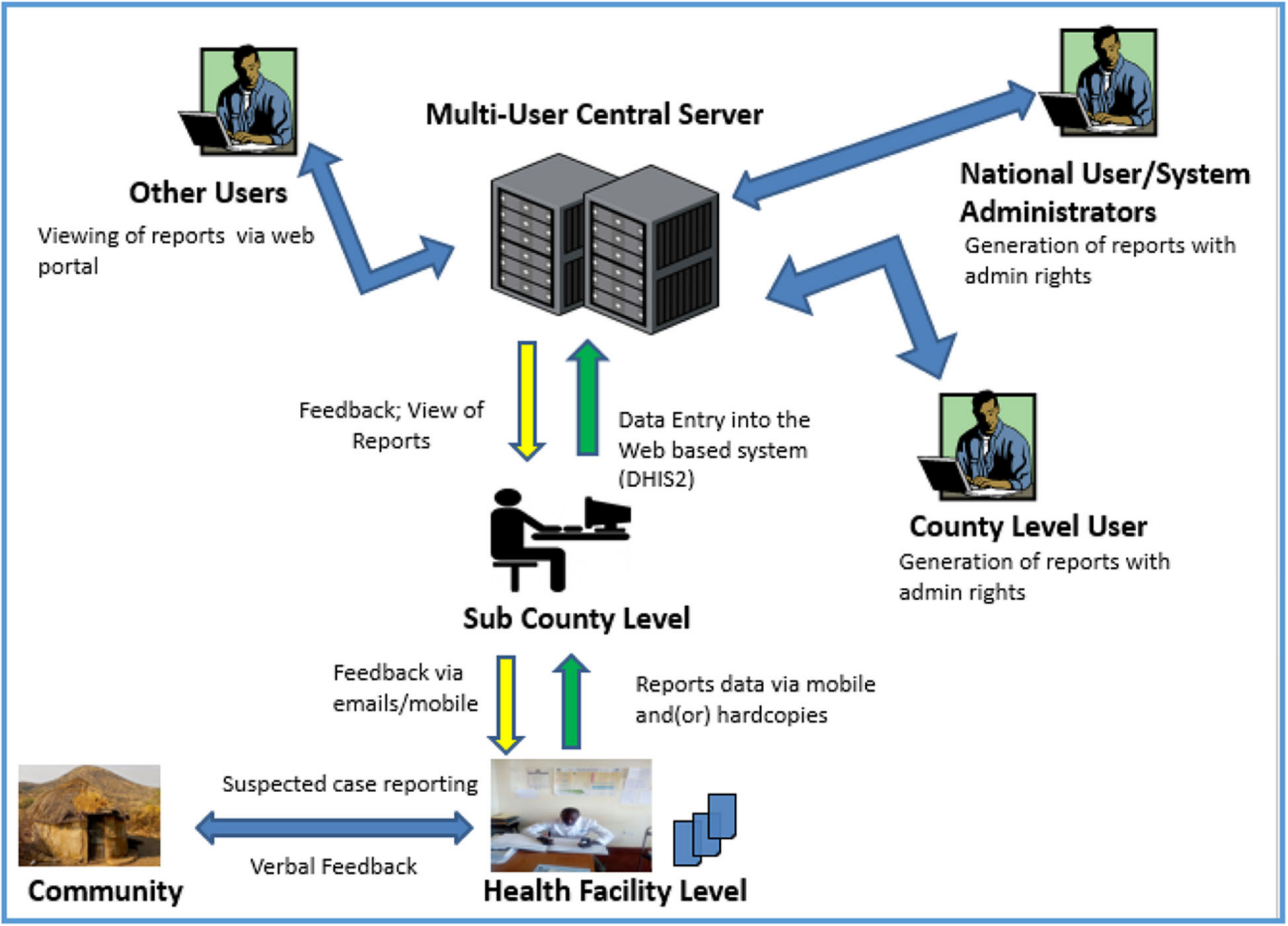
Surveillance data flow in Kenya. Surveillance data is sent by health facilities surveillance focal persons to sub county surveillance officer via a mobile phone sms or hardcopies. The surveillance data is then entered into the web based DHIS2 system by the sub county surveillance officer. Users at County, National and other organizations can now access the data in DHIS2. Source: Figure was generated by author David Kareko using Microsoft PowerPoint application. No special licenses or copy rights were required to use or publish

After the switch to DHIS2, the surveillance focal persons based in health facilities continued to send surveillance reports (events-based or weekly disease workload) via a short message service (SMS) to the sub county surveillance officers who would then enter the data into DHIS2. The county surveillance officers (County Surveillance Coordinators and County Health Record and Information Officers) and national surveillance officers would then access and monitor the data by accessing the DHIS2. The flow of the surveillance data in Kenya is shown in Fig. 1.

During the 1 month transition from eIDSR to DHIS2 in late 2016, completeness (proportion of health facilities submitting weekly reports) and timeliness of reporting plummeted from an average of 60 and 80% respectively to an average of 45 and 40%. respectively. The low reporting rates were attributed to inadequate training of surveillance officers on the new system. The MOH hypothesized that re-training surveillance officers would improve reporting rates and requested support to conduct a re-training of the county and sub county surveillance officers from counties where reporting rates were most affected (data entry is mainly done by sub county surveillance officers). Training was conducted between February and March 2017 by MOH with technical support from I-TECH Kenya. In this paper, we share the impact of training on timeliness and completeness of IDSR reporting rates in the new reporting platform. We also report on the challenges affecting the surveillance reporting rates at the various levels.

## Methods

### Study design

To determine how the use of DHIS2 surveillance technology impacted on the rates of timeliness and completeness of reporting of priority diseases in Kenya, we conducted a quasi-experimental study. To determine the counties to include in our sample of study, we applied the use of purposive sampling technique. The main characteristic that informed the selection of any given county for study was its rate of reporting (completeness and timeliness). Those counties with the lowest rates of reporting were therefore considered for the study. To completely cover the objectives, a mix of parametric and non-parametric tests was applied in respect to the given characteristics of the various data collected.

### Study setting

Kenya is divided into 47 counties and 300 sub counties. In February and March 2017, the Ministry of Health re-trained 78 surveillance officers (60 sub county surveillance coordinators, 10 county surveillance coordinators and 8 county health records and information officers) from 13 prioritized counties whose surveillance reporting rates (proportion of health facilities sending weekly reports) were the lowest. Training of other counties was to be done once more funding was available.

**Fig. 2:**
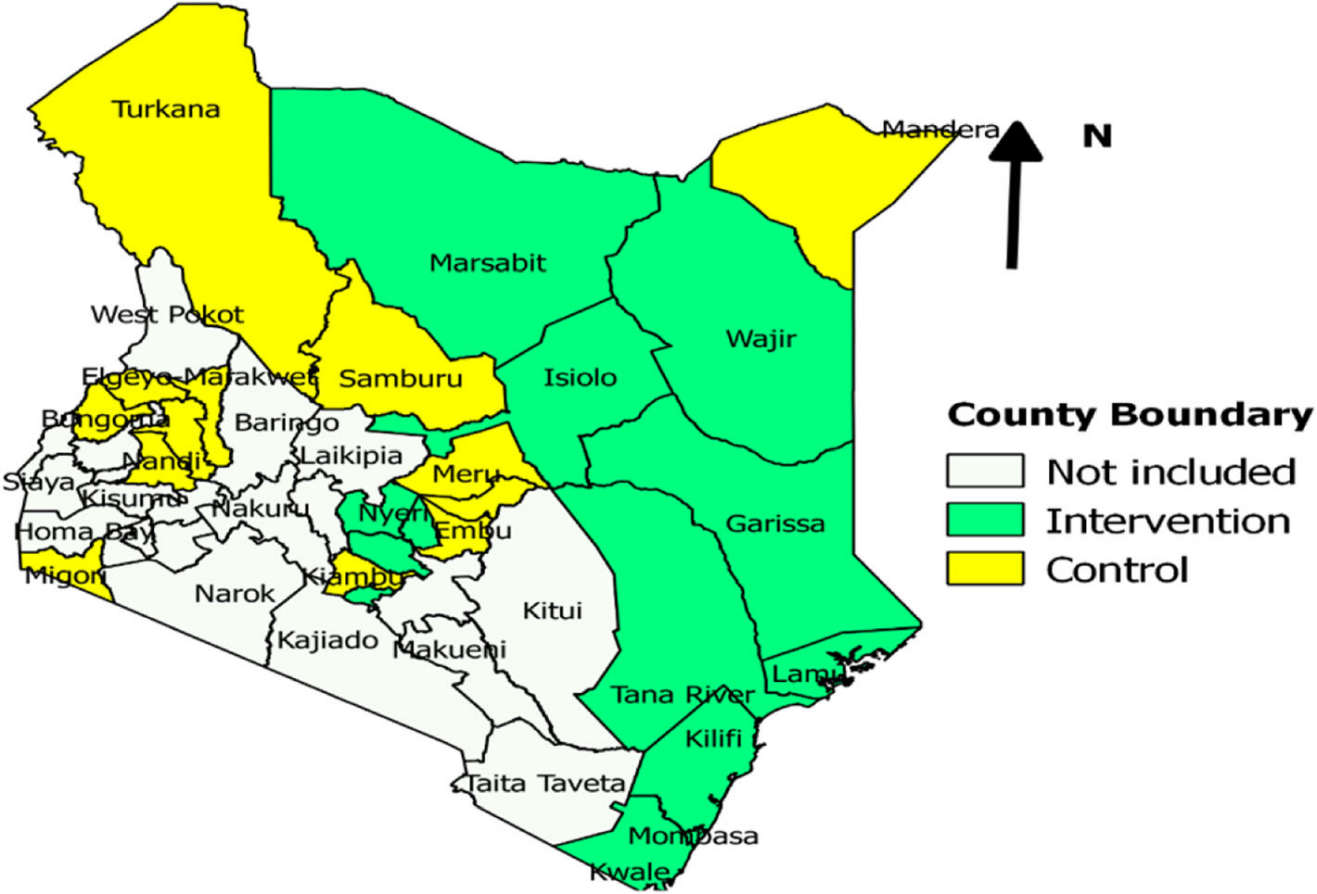
Map of Kenya showing 13 intervention and 13 control/comparison counties. *Intervention counties*: Mombasa, Kilifi, Kwale, Lamu, Tana River, Garissa, Wajir, Isiolo, Marsabit, Muranga, Nyeri, Kirinyaga, Nairobi. *Control/comparison counties*: Mandera, Vihiga, Kiambu, Tharaka Nithi, Elgeyo Marakwet, Meru, Turkana, Bungoma, Samburu, Migori, Nandi, Embu, Trans Nzoia. Source: Map was generated by author David Kareko using free QGIS software v2.2. No licenses were required to use or publish

From December 2016 to May 2017, we conducted an assessment of the surveillance training and its associated impact in the 13 selected counties (intervention counties). In addition, we also included in the assessment another 13 comparison counties (Fig. 2). An intervention county was defined as one who’s county and sub-county surveillance officers had received refresher training on DHIS2 in February or March 2017. These 13 intervention counties were also the poorest performing counties at an average weekly reporting rate (proportion of health facilities submitting weekly reports) of 45% over a 15 week reporting period from Epi Week 48 of 2016 to Epi Week 10 of 2017. A comparison county was defined as a one that belonged to the next 13 poorest reporting counties and that had not received the refresher training. The average reporting rate for the comparison counties was 49% over the same 15 week reporting period.

The 13 non-intervention counties were selected for comparison because they had the next poorest reporting rates and would therefore be a good comparison to the intervention counties as they would likely have similar challenges. The total number of health facilities at the beginning of the study was 1876 and 2439 in the intervention and comparison counties, respectively. However, this number varied slightly across the observation weeks mainly because some facilities were either added or dropped from the reporting list by the Ministry of Health.

### Training of participants (the intervention)

The intervention was a 2-day refresher training that was conducted in February and March 2017. The refresher training used the same curriculum that had been used during the initial training before the switch to DHIS2. However, the theory sessions were excluded, and the training focused solely on the practical elements of managing data in DHIS2. Specifically, the participants (county and sub-county disease surveillance focal persons) were trained on how to access the DHIS2 system through the web and mobile app, how to sign up and log in, how to enter surveillance data (indicator and event based) and how to analyze the data.

### Operational definitions

For both the intervention and comparison counties, completeness of reporting was defined as the proportion of health facilities that had weekly surveillance reports uploaded in DHIS2. One report was expected per facility per week. For each study group, timeliness of reporting was defined as the proportion of health facilities that uploaded weekly surveillance reports on time in DHIS2. A weekly surveillance report was considered to be on time if it was submitted by the subsequent Wednesday after the end of the reporting week.

Comparison of completeness and timeliness was made over 27 weeks’ period i.e.15 weeks before the intervention and 12 weeks after the intervention for both the intervention and comparison counties. Our target was to compare 15 weeks before and after the intervention but we had to limit the post intervention period to 12 weeks to guarantee that the comparison counties had not conducted any refresher training.

### Data management and analysis

#### Pre and post training test data

Before the commencement of the re-training, the 78 surveillance officers selected from the intervention counties were subjected to a structured test via Google form to assess their understanding and level of knowledge in using DHIS2 surveillance platform (See Additional file 1 for the pre/post-test). The same was done after the re-training process. The knowledge tested was majorly on the ability of the surveillance officer to access DHIS2, enter data, download data and analyze it as well as send surveillance reports using the DHIS2 platform. Their responses were analyzed and percentage scores ranging from 0 to 100% awarded and recorded for analysis. Since the recorded percentage scores involved results from two different samples (pre-test and post-test), we used the paired sample t-test for those who did both tests to determine if there was a significant change in the level of knowledge on the use of the DHIS2 surveillance platform before and after re-training of the surveillance officers.

#### Survey on challenges affecting surveillance

To understand the various challenges that affected the surveillance officers during reporting, we administered a structured questionnaire to the 78 participants from the 13 intervention counties (See Additional file 2 for the questionnaire). The questionnaire was administered via the Google form. The potential challenges covered in the questionnaire were assigned scores using the Likert scale that ranges from 0 to 5. Responses with a score below 3 were considered to be posing minor or no challenge while those that registered a score of 3 and above were viewed as posing major challenges (the higher the score the major the challenge). The Likert scores from the 78 participants were then averaged for each response and a graphical representation done to determine the most significant challenges that affected surveillance reporting.

#### Completeness and timeliness of reporting

Surveillance reporting data before and after re-training from each of the 26 counties (13 intervention counties and the other 13 comparison counties) was downloaded from the DHIS2 platform. The data was then aggregated week-wise, excluding any information or identifier relating to a specific individual. Analysis was pooled for both groups of counties (intervention and comparison counties) in order to increase the sample size and power of the study.

To determine the rate of change in completeness and timeliness of surveillance reporting, we incorporated the use of frequency distributions to calculate the percentage rates before and after training for both the intervention and comparison counties. We then used the chi square test of independence to determine whether there was any significant difference in the rate of reporting surveillance before and after training. Our decision to use the Chi square test of independence was informed by the fact that our data involved frequencies and it also did not exhibit a normal distribution.

To analyze the trends on completeness and timeliness of reporting, we used the whole weekly reports of the 23 diseases that were supposed to be reported with no consideration on the type of condition or disease being reported in the reports (zero reporting was the government policy for diseases that had not been observed in any given week). For a report to be considered timely, it had to have been received by Wednesday of each week.

The data was collected over a 27 weeks period, the first 15 weeks categorized as weeks before training while the remaining 12 weeks were categorized as weeks after training.

## Results

### Pre and post test

A total of 78 surveillance officers (60 sub county surveillance coordinators, 10 county surveillance coordinators and 8 county health records and information officers) from 13 counties and 62 sub counties were trained. Out of the 78 officers, 43 completed the pre- test and 74 completed the post- test. Training was done in 3 groups and one group did not do the pre- test due to logistical challenges related to electronic forms. For the 43 who did both tests, the average score for the pre-test was 58% (range 32–89%) and this increased to 74% (range 58–100%) in the post-test. The difference in mean between the 2 groups for those who did both tests was 17 with a t-score of 7.81 and *p*-value < 0.00005 (two tailed paired t-test at 0.05 significance level).

### Completeness of reporting

**Fig. 3:**
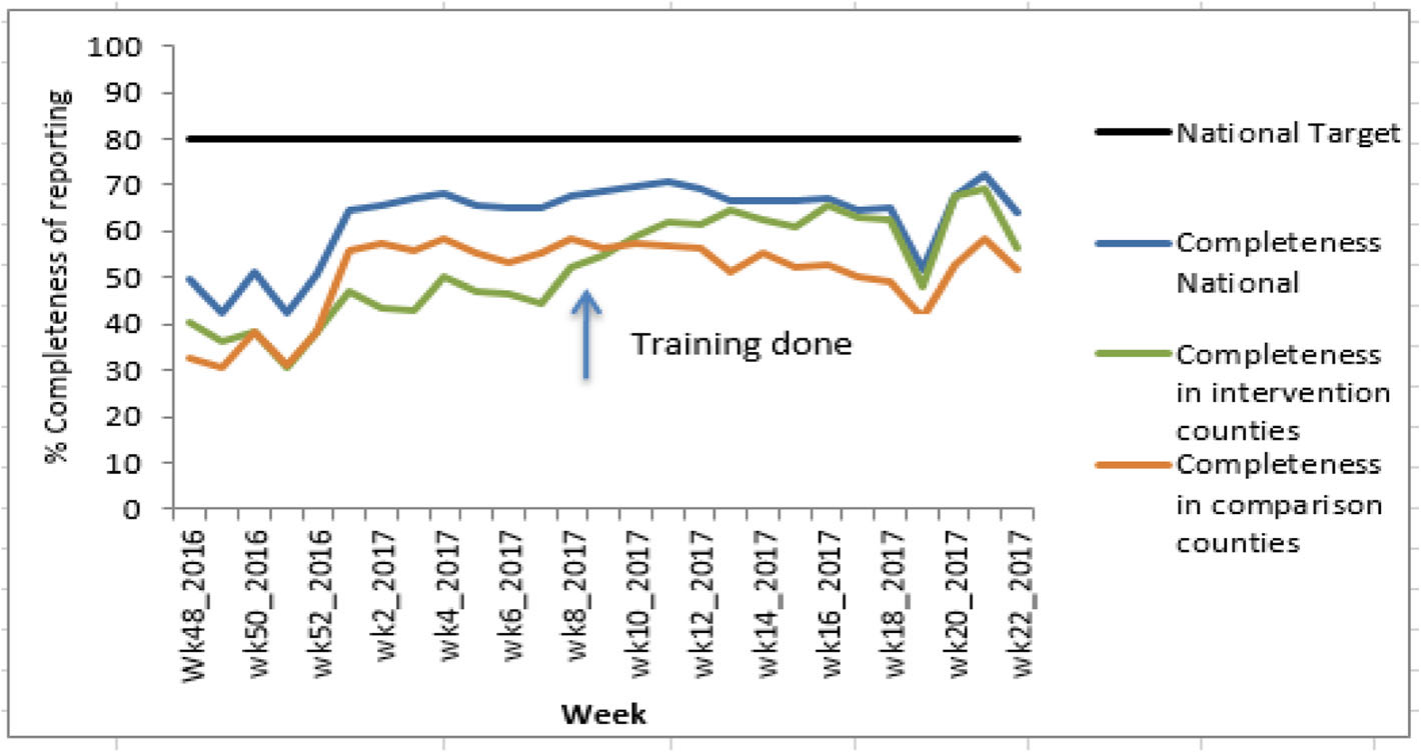
Trends in completeness of reporting pre and post training

After the training, the trend for the completeness of reporting for the intervention counties gradually improved from Epi week 9 of 2017 and surpassed the comparison group (Fig. 3). Additionally, the trend for completeness of reporting for the intervention group neared the national completeness of reporting after 7 weeks. There was a decline of reporting rates in week 19 of 2017 for all the groups due to an unexpected system downtime but this improved again in the subsequent week. However, the reporting rates for the intervention group remained higher than the comparison group in all the weeks of observations (Fig. 3).

Overall, after the training the average completeness of reporting for the intervention counties increased from 45 to 62%, that is by 17 percentage points (95% CI 16.14–17.86; *P* value < 0.0001) as shown in Table 1. The average completeness of reporting across comparison groups increased from 49 to 52%, that is by 3 percentage points (95% CI 2.23–3.77**;***P* value < 0.0001**)**. Despite this improvement of the intervention group after training, the reporting rates for all groups remained lower than the expected national target of 80% (Fig. 3).

**Table 1:** Completeness of surveillance reporting pre and post training

	Intervention counties			Comparison counties		
	Number of facility weekly reports received (a)	Number of expected facility weekly reports (b)	% Reporting rate (a/b × 100)	Number of facility weekly reports received (c)	Number of expected facility weekly reports (d)	% reporting rates (c/dx100)
**before training** (15 weeks period)	12,716	28,352	45%	18,023	36,769	49%
**after training** (12 weeks period)	14,011	22,579	62%	15,332	29,290	52%
Difference before and after training			**17%** (95% CI 16.14–17.86)			**3%** (95% CI 2.23–3.77)
Chi square			1491			72
*P* value			< 0.0001			< 0.0001

The difference between the change of 17% in the intervention group and 3% in the comparison group is 14% and part of this can be attributed to the intervention

Notes on Table 1: The reduction in the number of expected weekly health facility reports before and after training is due to the difference in time period i.e. the number of weeks allocated for each category. There are 12 weeks of observation allocated after training and 15 weeks before training

### Timeliness of reporting

**Fig. 4:**
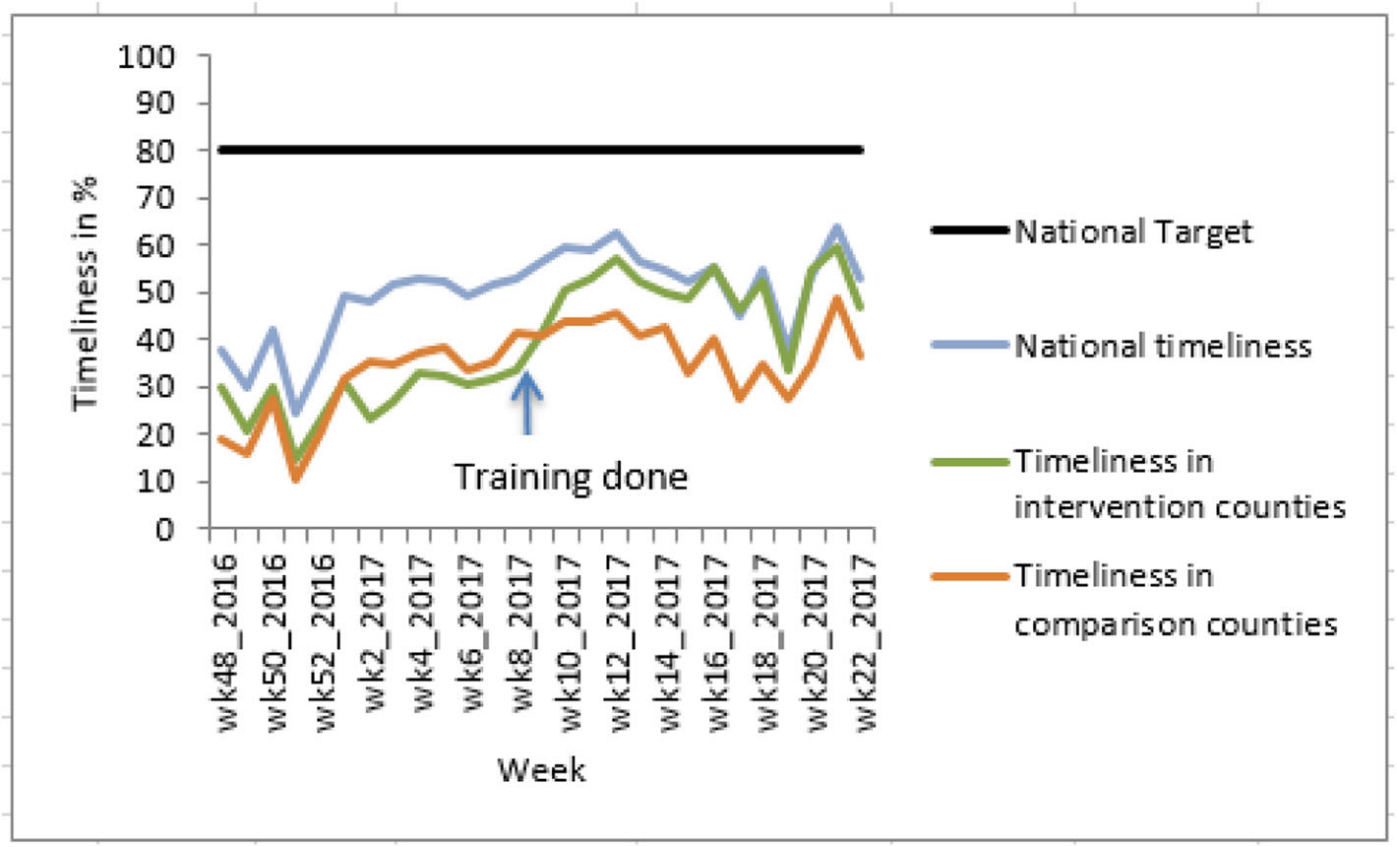
Trends in timeliness of surveillance reporting pre and post training

We observed an almost similar trend for the timeliness as for completeness. The timeliness trend for the intervention group improved markedly after the training as compared to the comparison group (Fig. 4). The trend for timeliness of reporting for the intervention group also matched the national average reporting rate after about 7 weeks after the training. The timeliness of reporting was noted to have improved more than completeness.

Overall, the average timeliness of reporting increased after the training from 30 to 51% i.e.by 21 percentage points (95% CI 20.16–21.84; *P* value < 0.0001) for the intervention group as compared to an increase from 31 to 38% for the comparison group i.e.by 7 percentage points (95% CI 6.27–7.73; *P* value < 0.0001) (Table 2). Despite this improvement, the reporting rates for all groups remained lower than the national target of 80% (Fig. 4).

**Table 2:** Timeliness of surveillance reporting pre and post training

	Intervention counties			Comparison counties		
	Number of weekly reports received (a)	Number of expected weekly reports (b)	% Timeliness (a/b ×100)	Number of weekly reports received (c)	Number of expected weekly reports (d)	% Timeliness (c/d ×100)
**before training** (15 weeks period)	8572	28,352	30%	11,435	36,769	31%
**after training** (12 weeks period)	11,490	22,579	51%	11,164	29,290	38%
Difference before and after training			**21%** (95% CI 20.16–21.84)			**7%** (95% CI 6.27–7.73)
Chi square			2247			357
*P* value			< 0.0001			< 0.0001

The difference between the change of 21% in the intervention group and 7% in the comparison group is 14% and part of this can be attributed to the intervention

### Challenges affecting reporting rates

**Fig. 5:**
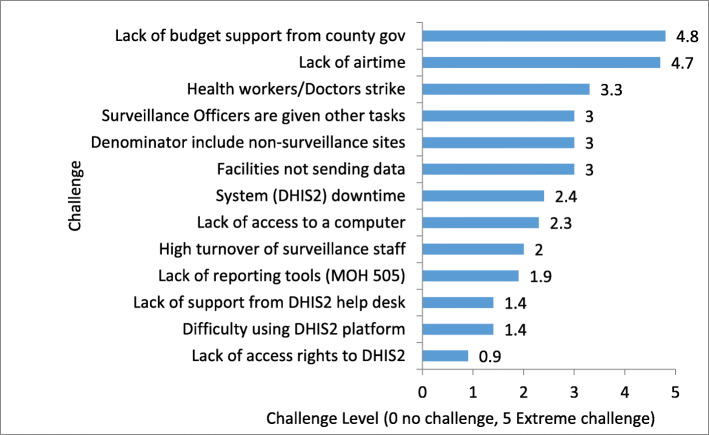
Challenges affecting surveillance reporting rates (*n* = 78 responders). Interpretation: Score < 3: Minor or no challenge; Score = > 3: Major challenge

From the responses obtained from the administered questionnaires, the challenges affecting the reporting rates were identified. The major challenges (score of 3 and above) that the surveillance officers reported were issues such as lack of supporting budget from the county governments (score 4.8), lack of airtime for reporting (score 4.7), health workers/doctors strikes (score 3.3), surveillance officers having other competing tasks (score 3.0), reporting denominator includes non-surveillance sites (score 3.0) and health facilities not sending data to the sub county (score 3.0). A surprise finding of this survey was that technical challenges related to use of DHIS2 that the training was supposed to solve had a very low score of 1.4 and therefore not considered a major challenge. The full list of challenges and scores is as shown in Fig. 5.

## Discussion

Many countries in the world are now using electronic systems for public health surveillance reporting as these have been shown to improve reporting rates [19–23]. The District Health Information System (DHIS2) is one of the most popular electronic systems for reporting health related data and is currently used in over 40 countries [24]. DHIS2 provides an opportunity to embrace a sustainable open source technology to improve public health surveillance reporting. However, like any other health information system, resources such as computers, internet and training opportunities must be provided to promote its optimal implementation [25].

Kenya migrated IDSR reporting from a standalone web-based surveillance system to the robust DHIS2 system in 2016 and this provided an opportunity to observe and learn from the transition. When countries embrace use of electronic systems for reporting, it is common for health personnel to blame inadequate capacity building for poor performance of indicators. While there is no doubt that training can improve reporting rates in general as shown in some studies [26–28], this paper tries to quantify to what extent the improvement can be attributed to training by using a comparison group. The study also tries to show the improvement trajectory and how long it can last. This is important so that program managers are aware that training can only improve surveillance reporting indicators to a certain extent beyond which other limiting factors or challenges must be identified and addressed.

Our study demonstrated that the refresher training improved knowledge on use of DHIS2 surveillance platform which eventually led to better outcomes in improving surveillance reporting rates. In terms of the actual outcomes of the training, the completeness of reporting increased by 17 percentage points in the intervention group compared to only 3 percentage points in the comparison group. The difference between the increases in the two groups was therefore 14 percentage points. Likewise, the timeliness of reporting improved by 21 percentage points in the intervention group compared to 7 percentage points for the comparison group. Therefore, the difference between the increases in the two groups was also 14 percentage points. These increases were most likely due to the re-training, although rates were increasing in both groups somewhat before the intervention.

Another key observation made in this study was that reporting rates in the intervention counties improved almost immediately after the training; it took only 7 weeks for the reporting rates in the intervention group to match the national average reporting rates (which were higher) after which the rates seemed to stagnate at between 60 and 70% for completeness and below 60% for timeliness. This therefore indicates that despite the training impact, there are likely other factors other than the training affecting the reporting rates. One of the objectives of this paper was therefore to try and find out what these other limiting factors or challenges could be.

There are many challenges that affect surveillance reporting rates and training is often wrongly seen as a quick fix to the poor reporting rates. From the findings in this study, it was clear that training was only a partial solution but that other challenges affecting surveillance must be addressed by the government and other stakeholders if reporting rates are to improve further towards the desired target of 80%.

As noted in our study, the training on use of DHIS2 reporting platform addressed some challenges such as inadequate technical capacity in using DHIS2. However, challenges related to use of DHIS2 were not really perceived by users as being the biggest hindrance to system use and complete and timely reporting. The major deterrents to system use and better reporting rates were other challenges such as lack of budgetary support and lack of airtime for reporting which could not be resolved through the training we conducted.

While most of the challenges we found are quite similar to many other countries implementing IDSR in the African continent, our study was unique in that it quantified and ranked the challenges in order to make it easier for the program managers to prioritize interventions. Some of the challenges found in other countries such as Zambia, Ghana, Malawi, Uganda, Democratic Republic of Congo and others includes: inadequate trained staff, high turnover of staff, inadequate funding, poor coordination, poor infrastructure, lack of technical guidelines and standard case definitions, inadequate supervision/feedback and low prioritization of surveillance activities by health workers. Others include information technology challenges such as limited access to computers and internet and inadequate technical support [12–15, 27, 29, 30].

## Limitations

This study’s limitations included use of reporting rates data for the pre intervention period (period before training) that was collected during a nationwide doctors’ strike. Though the strike only affected doctors and not other cadres like surveillance officers, it is likely that this may have had a negative impact on the reporting rates. However, as this was a nationwide strike, we believe that any effects on the reporting rates would have affected equally the intervention and comparison groups. Secondly, the study observation period was limited to a short time (15 weeks pre-training and 12 weeks post-training) as this is the period that no similar training intervention occurred in any of the comparison counties. This may have affected the opportunity to see how the reporting rates would vary in the long run and in different contexts. Thirdly, the comparison counties were selected as they were the closest to the intervention counties in terms of poor performance and hence the best for comparison. However, their reporting rates were slightly better than the intervention counties and hence the opportunity to improve may not have been the same as in the intervention counties.

## Conclusions

Public health surveillance reporting is crucial for timely detection and response to disease outbreaks. With support from governments and other strategic partnerships such as the Global Health Security Agenda and World Health Organization, many countries have made good progress in improving public health surveillance systems. Surveillance reporting indicators therefore continue to improve especially through adoption of electronic systems. In this paper, we have demonstrated that training on the use of surveillance reporting platforms has some role to play in improving the reporting indicators. However, there are other additional and equally important challenges that affect surveillance reporting systems and a systematic evaluation of the surveillance system must be conducted regularly in order to identify and address them.

In trying to address some of these challenges in the African countries, the World Health Organization released the 3rd edition of IDSR technical guidelines and training modules in 2019. These guidelines are a great improvement from earlier editions and includes a new module on electronic IDSR and how this can be implemented by countries. There is also great emphasis on training IDSR at both pre-service (universities and colleges) and in-service (on job training) level to ensure that knowledge of IDSR is universally available and hence addressing some of the challenges like inadequately trained staff and high turn-over of staff. Therefore, if these new IDSR technical guidelines are adapted by countries, there should be some great improvement in surveillance systems [31]. Other challenges especially those related to inadequate resources will require a systematic approach and commitment by the governments to address them.

## Supplementary information

**Additional file 1.** This is the questionnaire that was used as the pre and post-test before and after the training that was conducted on use of District Health Information System 2(DHIS2). The questionnaire were the same for both tests but only the pre-test has been attached here.

**Additional file 2.** This is the questionnaire that was used to collect data on the challenges affecting surveillance in Kenya.

## Data Availability

The datasets used and/or analysed during the current study are available from the corresponding author on reasonable request.

## Abbreviations

CDC: Centers for Disease Control and Prevention
DHIS2: District Health Information System 2
e-IDSR: electronic Integrated Disease Surveillance and Response
FAO: United Nations’ Food and Agriculture Organization
GHSA: Global Health Security Agenda
IDSR: Integrated Disease Surveillance and Response
IHR: International Health Regulations
I-TECH Kenya: International Training and Education Centre for Health Kenya
LMIC: Low and Middle Income Countries
MOH: Ministry of Health
OIE: International Organization for Animal Health
OHI: One Health Initiative
SMS: Short Message Service
WHO: World Health Organization
