# Supplementary material for: Use of technology for public health surveillance reporting: opportunities, challenges and lessons learnt from Kenya
Source: BMC Public Health. 2020 Jul 13;20:1101. doi: 10.1186/s12889-020-09222-2 (PMC7359619; doi:10.1186/s12889-020-09222-2)
Supplement: Supplementary file 1 — Additional file 1. This is the questionnaire that was used as the pre and post-test before and after the training that was conducted on use of District Health Information System 2(DHIS2). The questionnaire were the same for both tests but only the pre-test has been attached here. [file 12889_2020_9222_MOESM1_ESM.pdf]

DHIS2 Training Pre-test

Answer all questions

* Required

1. Sub County name: * ____________________

2. Designation * *Mark only one oval.*

   - ( ) Health Records and Information officer
   - ( ) Surveillance Coordinators

3. The following can be used to access DHIS2 except? * *Check all that apply* 2 points

   - [ ] Internet connectivity
   - [ ] Computer
   - [ ] Smart phone
   - [ ] Tablet
   - [ ] Hospital registers
   - [ ] MFL code
   - [ ] Access rights

4. The following are DHIS2 functionalities * *Check all that apply.* 4 points

   - [ ] Data collection
   - [ ] Data collation
   - [ ] Data analysis
   - [ ] Case based reporting
   - [ ] Report generation
   - [ ] ICD 10 coding

5. DHIS2 IDSR reporting modules include * *Check all that apply.* 4 points

   - [ ] Event capture
   - [ ] Weekly reporting
   - [ ] Immediately reportable disease Public
   - [ ] Health Events

6. The following is true regarding organization units in DHIS2 except * *Mark only one oval.* 2 points

   - ( ) Include county, sub county ward and facility only
   - ( ) They represent reporting administrative hierarchy
   - ( ) Health facilities can be updated by the SCHRIO at the sub county level

7. Regarding Information on health facilities and Community Units in DHIS2, which of the following is most accurate? * *Mark only one oval.* 2 points

- MFL code, ownership, type and Geo codes
- MFL code, ownership and Geo codes
- MFL code, ownership, type, level and Geo codes
- MFL code, ownership, type and level

8. The following applications can be used for surveillance data management on DHIS2 *

Check all that apply. 7 points

- [ ] Data entry
- [ ] Event capture
- [ ] Pivot tables
- [ ] Data visualizer
- [ ] GIS
- [ ] Event Visualizer
- [ ] Report

9. The data set report MOH 505 aggregates data by? * *Mark only one oval.* 3 points

- Monthly
- Weekly
- Quarterly
- Daily

10. Data on the following data sets should be consistent on DHIS2 * *Check all that apply.* 4 points

- [ ] MOH 505
- [ ] MOH 105
- [ ] MOH 205
- [ ] MOH 705

11. How often should the surveillance coordinator compare their data with 2 points that of the HRIO * *Mark only one oval.*

- Weekly
- Monthly
- Not at all

12. The Kenya Wildlife Service Ranger in your area has notified the sub county of 15 cases of abortion by Zebras in Tsavo West National park. how would you report this information on DHIS2? * 4 points

13. You have received reports from one of your facility of 10 cases of measles in class 8 pupils and one death of a 2-year-old at home, six adults are admitted at the sub county hospital. How would you report this on DHIS2? * 4 points
