# Supplementary material for: Use of technology for public health surveillance reporting: opportunities, challenges and lessons learnt from Kenya
Source: BMC Public Health. 2020 Jul 13;20:1101. doi: 10.1186/s12889-020-09222-2 (PMC7359619; doi:10.1186/s12889-020-09222-2)
Supplement: Supplementary file 2 — Additional file 2. This is the questionnaire that was used to collect data on the challenges affecting surveillance in Kenya. [file 12889_2020_9222_MOESM2_ESM.pdf]

Challenges affecting surveillance reporting rates at the sub county level

* Required

1. County Name *

____

2. Sub County Name *

____

3. Designation of respondent

Check all that apply.

- [ ] County Disease Surveillance Coordinator
- [ ] Sub County Disease Surveillance Coordinator
- [ ] County Health Records and Information Officer Other:
  - [ ] ______________________________

4. Are you responsible for reporting surveillance data in your sub county ? *

Check all that apply.

- [ ] Yes
- [ ] No

On a scale of 0-5 please indicate how much the following challenges are responsible for the reporting rate performance in your sun county in the last 3 month (0 is not a challenge at all; 5 is a big challenge)

5. 1. Lack of access rights to DHIS2 * *Mark only one oval.*

0	1	2	3	4	5
⬭	⬭	⬭	⬭	⬭	⬭

6. 2. Difficulty using DHIS2 reporting platform * *Mark only one oval.*

0	1	2	3	4	5
⬭	⬭	⬭	⬭	⬭	⬭

7. 3. Lack of airtime * *Mark only one oval.*

0	1	2	3	4	5
⬭	⬭	⬭	⬭	⬭	⬭

8. 4. Lack of reporting tools (MOH 505) * *Mark only one oval.*

0	1	2	3	4	5
⬭	⬭	⬭	⬭	⬭	⬭

9. 5. Lack of access to a computer * *Mark only one oval.*

0	1	2	3	4	5
⬭	⬭	⬭	⬭	⬭	⬭

10. 6. Facilities not sending data *

Mark only one oval.

0 1 2 3 4 5

11. 7. Denominator include non-surveillance sites *

Mark only one oval.

0 1 2 3 4 5

12. 8. High turnover of surveillance staff *

Mark only one oval.

0 1 2 3 4 5

13. 9. Sub County Surveillance Officers are given other task *

Mark only one oval.

0 1 2 3 4 5

14. 10. Lack of budget support from county government *

Mark only one oval.

0 1 2 3 4 5

15. 11. Health workers/Doctors strike *

Mark only one oval.

0	1	2	3	4	5
⬭	⬭	⬭	⬭	⬭	⬭

16. 12. Lack of support from DHIS2 help desk *

Mark only one oval.

0	1	2	3	4	5
⬭	⬭	⬭	⬭	⬭	⬭

17. 13. System (DHIS2) downtime *

Mark only one oval.

0	1	2	3	4	5
⬭	⬭	⬭	⬭	⬭	⬭

18. 14. Others (specify) *
